# Dermatome‐Specific Herpes Zoster Following Corticosteroid Therapy for Lumbar Disc Herniation: A Case Report Illustrating the Immunocompromised District Theory

**DOI:** 10.1002/ccr3.73475

**Published:** 2026-09-09

**Authors:** Jun Liu, Lili Luo, Ming Zhou, Weifeng Chen, Na Cao, Zhanglin Pu, Yunwei Niu, Jiachen Guo, Huayuan Liao, Yi Li, Jun Gao, Kang Wei

**Affiliations:** ^1^ Department of Orthopedics Changzhou Hospital Affiliated to Nanjing University of Chinese Medicine Changzhou Jiangsu Province People's Republic of China; ^2^ Department of Rehabilitation Changzhou Hospital Affiliated to Nanjing University of Chinese Medicine Changzhou Jiangsu Province People's Republic of China

**Keywords:** case report, diagnosis, herpes zoster, immunocompromised district theory, lumbar disc herniation, pain phenotype

## Abstract

Systemic corticosteroids frequently utilized to manage lumbosacral radiculopathy introduce an immunological paradox by potentially triggering varicella‐zoster virus reactivation. While well‐documented in severely immunocompromised populations, the specific neuro‐immunological mechanisms in otherwise immunocompetent patients with lumbar disc herniation remain underappreciated. This case presents an observation conceptually relevant to the “Immunocompromised District” theory, proposing a theoretical link between acute radicular compression and a localized vulnerability that may correlate with the precise anatomical distribution of a viral outbreak. A 52‐year‐old male presented with acute mechanical right‐sided lumbar and anterior thigh pain. Magnetic resonance imaging revealed an acute L3‐L4 disc herniation compressing the L4 nerve root, coexisting with an asymptomatic chronic L4‐L5 herniation. Four days after initiating intravenous dexamethasone, the patient experienced a sudden phenotypic shift in symptoms: the mechanical dullness transformed into severe, electric‐shock‐like neuropathic pain. This heralded a herpes zoster eruption remarkably confined to the acutely symptomatic L4 dermatome, completely sparing the chronically compressed L5 region. Following corticosteroid discontinuation and initiation of antiviral therapy, the lesions resolved within 1 week, leaving mild post‐herpetic neuralgia at the one‐month follow‐up. Given the lack of objective molecular data, we hypothesize a multifactorial pathogenesis for this phenomenon. A theoretical “double hit” mechanism—comprising corticosteroid‐induced systemic immunosuppression and localized vulnerability from acute radiculopathy—may potentially facilitate viral reactivation in a susceptible host (e.g., possessing age‐related and metabolic risk factors). Clinically, a sudden qualitative shift in pain phenotype from mechanical to neuropathic during steroid therapy for lumbar degeneration may serve as an early clinical clue of impending herpes zoster. Recognizing this dynamic prevents misinterpreting viral prodromes as mechanical exacerbations, thereby avoiding the detrimental escalation of immunosuppressive therapy.

## Key Clinical Message

Herpes zoster may emerge during systemic corticosteroid treatment for lumbar disc herniation. A sudden shift from mechanical to neuropathic pain may provide a critical early diagnostic clue, potentially preventing misdiagnosis and the inappropriate escalation of immunosuppression.

## 1 Introduction

Lumbosacral radiculopathy arising from lumbar disc herniation (LDH) persists as one of the most clinically challenging sources of disability encountered in spinal practice. Systemic corticosteroids are sometimes used in the conservative management of acute lumbosacral radiculopathy, leveraging their dual capacity to mitigate acute inflammatory cascades and stabilize hyper‐excitable neuronal membranes [1, 2]. However, the use of this therapeutic strategy introduces an inherent immunological dilemma: the same mechanism intended to mitigate sterile radicular inflammation can simultaneously induce a systemic attenuation of cell‐mediated immunity (CMI). This dual effect introduces a specific vulnerability regarding the Varicella‐Zoster Virus (VZV). Sequestered in a latent state within the dorsal root ganglia (DRG), the virus relies on robust immune surveillance to maintain dormancy; consequently, iatrogenic immune dampening can disrupt this delicate virus‐host equilibrium. In patients with preexisting baseline vulnerabilities, this pharmacological suppression may facilitate viral reactivation, serving as a contributing factor rather than a singular trigger [3].

While the association between corticosteroid administration and Herpes Zoster (HZ) is well‐established in severely immunocompromised cohorts—such as organ transplant recipients or oncology patients—the specific neuro‐immunological mechanisms governing this phenomenon in otherwise immunocompetent LDH patients remain largely underappreciated. To bridge this gap, Ruocco's concept of the “Immunocompromised District (ICD)” offers a valuable theoretical framework. This theory posits that nerve compression implies more than a disruption of axoplasmic transport; it fundamentally isolates the affected segment, blockading local neuro‐immune crosstalk [4, 5]. Consequently, we hypothesize that the dermatome innervated by the compressed root may act as a locus minoris resistentiae—a localized microenvironment with potentially compromised immune defense. Furthermore, clinical observations suggest that the degree of this local vulnerability may correlate with inflammatory intensity: acute, severe radiculitis might be associated with a more pronounced local immune alteration than chronic, stable compression [6, 7]. It is within this vulnerable landscape that the “double hit” occurs: when the systemic immunosuppression of corticosteroid therapy is superimposed upon this localized fragility, the virus opportunistically reactivates, preferentially targeting the segment of most intense inflammation [8, 9]. This pathological synergy assumes a particularly deceptive guise in high lumbar disc herniations (High LDH, L1–L3). Although accounting for a mere ~5% of lumbar pathologies [10, 11], High LDH presents a unique diagnostic challenge due to its distribution: the resulting femoral neuralgia creates a profound “symptomatic mimicry” with the prodromal pain of L3–L4 zoster [12, 13]. This overlap creates a significant diagnostic challenge: clinicians may easily conflate the viral prodrome with an exacerbation of the primary spinal pathology and, inadvertently, escalate steroid dosage—thereby exacerbating viral replication and accelerating the subsequent outbreak.

Herein, we document a case of a patient presenting with an MRI‐confirmed acute high lumbar disc herniation at the L3–L4 level (the symptomatic lesion), coexisting with an incidental, chronic degenerative herniation at L4–L5. The clinical sequelae following corticosteroid therapy were particularly revealing: the resultant Herpes Zoster (HZ) outbreak manifested a striking anatomical‐pathological gradient. Specifically, the vesicular rash was densely consolidated within the acutely compromised L4 dermatome, yet only sparsely scattered across the chronically involved L5 territory. By delineating this precise correlation—where the intensity of viral reactivation mirrors the severity of the underlying neural injury—this report aims to dissect the synergistic pathology of structural compression and pharmacologic immunosuppression. Ultimately, our analysis seeks to arm clinicians with the critical foresight needed to distinguish prodromal viral neuralgia from mechanical exacerbation, facilitating earlier and more accurate differential diagnosis.

## 2 Case Presentation

### 2.1 Patient Profile and Clinical Findings

A 52‐year‐old male was admitted to our service reporting the acute onset of lumbar pain radiating distinctly into the right buttock and anterior thigh. While the symptoms had initiated 4 days prior to presentation, the patient described a rapid crescendo in severity over the preceding 72 h. Characterized as a continuous, deep, distending ache—rating a 7 out of 10 on the Visual Analog Scale (VAS)—the pain exhibited a clear mechanical component, intensifying significantly with physical exertion. His background history was significant for Type 2 Diabetes Mellitus, currently managed with oral metformin. Pertinent to the subsequent clinical course, he recalled a childhood episode of varicella (chickenpox) but explicitly denied recent trauma, febrile prodromes, or any known immunodeficiency syndromes.

On physical examination, the lumbar spine showed marked restriction in range of motion, with palpation eliciting distinct tenderness at the right paravertebral L3–L4 level. The provocation maneuvers provided critical localization differentiating high from low lumbar pathology: the patient exhibited a positive Femoral Nerve Stretch Test (FNST) on the right, which faithfully reproduced the hallmark distending pain of the anterior thigh. In contrast, the Straight Leg Raise (SLR) test remained negative—restricted only by constitutional hamstring tightness. This clinical dissociation was further corroborated by neurological assessment, which revealed a diminished right patellar reflex (L4 arc) relative to the contralateral side, whereas bilateral Achilles tendon reflexes remained intact. At this initial juncture, however, no gross motor weakness or objective sensory deficits were detectable.

### 2.2 Radiological Assessment and Laboratory Results

**FIGURE 1:**
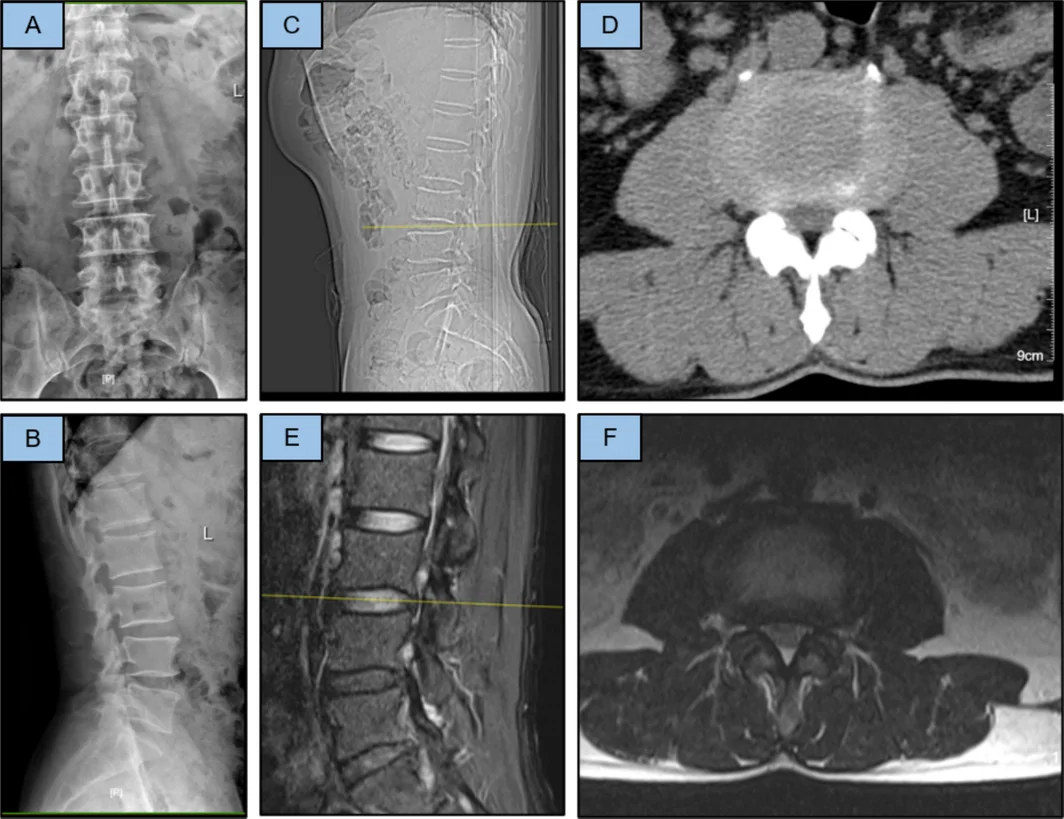
*Pre‐treatment radiological assessment of the lumbar spine*. (A, B) Anteroposterior and lateral radiographs showing mild lumbar spondylosis. (C) CT scout view indicating the L3–L4 axial scan plane (yellow line). (D) Axial CT at L3–L4 showing disc bulging and facet hypertrophy. (E) Sagittal T2‐weighted MRI. The symptomatic L3–L4 level (yellow line) shows mild degeneration (Pfirrmann Grade II‐III), contrasting with the severely degenerated L4‐L5 “black disc” (Pfirrmann grade IV). (F) Axial MRI at L3–L4 revealing a right paracentral disc protrusion. This causes mild‐to‐moderate lateral recess stenosis, directly impinging on the traversing right L4 nerve root.

Lumbar MRI revealed a notable radiologic‐clinical mismatch. The clinically symptomatic L3‐L4 segment showed mild degeneration with relatively preserved hydration (Pfirrmann Grade II–III). However, a focal right paracentral disc protrusion caused mild‐to‐moderate lateral recess stenosis, directly impinging on the traversing right L4 nerve root (Figure 1) and providing an anatomical substrate for the radicular pain.

Conversely, the L4‐L5 segment exhibited severe degeneration, presenting as a dehydrated “black disc” with distinct height loss (Pfirrmann Grade IV) and a broad‐based protrusion. Despite worse overall structural deterioration, it lacked focal neural compression, acting merely as an asymptomatic bystander.

Notably, while MRI confirms the anatomical mechanical impingement at L3–L4, it cannot quantify local inflammation or immune alterations. Therefore, linking this specific mechanical compression to the subsequent zoster reactivation remains an observational hypothesis rather than a radiological certainty.

### 2.3 Therapeutic Intervention and Clinical Evolution

To address the acute radicular inflammation, a targeted therapeutic regimen was promptly initiated, comprising intravenous dexamethasone (10 mg daily for a 3‐day course, yielding a total cumulative dose of 30 mg) to mitigate nerve root edema. This was supplemented by oral enteric‐coated diclofenac sodium (75 mg once daily for exactly 3 days) for multimodal analgesia. Initially, the response was encouraging: by the third day of therapy, the patient's primary complaint—the distending mechanical pain in the lower back and thigh—had significantly abated, with the VAS score retreating to a manageable 3/10.

On the fourth day, the patient's clinical presentation altered significantly. He reported a sudden change in his symptom profile: the previous dull, mechanical pain was replaced by new‐onset, continuous burning pain. This was accompanied by paroxysmal, electric‐shock‐like neuropathic pain radiating precisely along the anterior right thigh and medial lower leg. These subjective sensory changes corresponded with objective cutaneous findings: physical examination demonstrated an early cluster of erythematous papules and vesicles strictly confined to the right L4 dermatome (spanning from the anteromedial thigh to the medial calf). Given this classic clinical presentation—a unilateral dermatomal eruption combined with distinct neuropathic pain—a clinical diagnosis of herpes zoster was made. This initial assessment was corroborated by a prompt dermatology consultation. The specialist noted that the characteristic dermatomal topography effectively differentiated this presentation clinically from other mimicking vesicular dermatoses, such as zosteriform herpes simplex virus (HSV) infection or localized contact dermatitis. However, we must explicitly acknowledge that this diagnosis remained purely clinical. Objective virological confirmations—such as polymerase chain reaction (PCR) testing of the vesicle fluid for VZV DNA, viral culture, or specific serological testing—were not performed in this setting.

### 2.4 Outcome and Follow‐Up

Corticosteroids were immediately discontinued. An antiviral protocol (valacyclovir).

**FIGURE 2:**
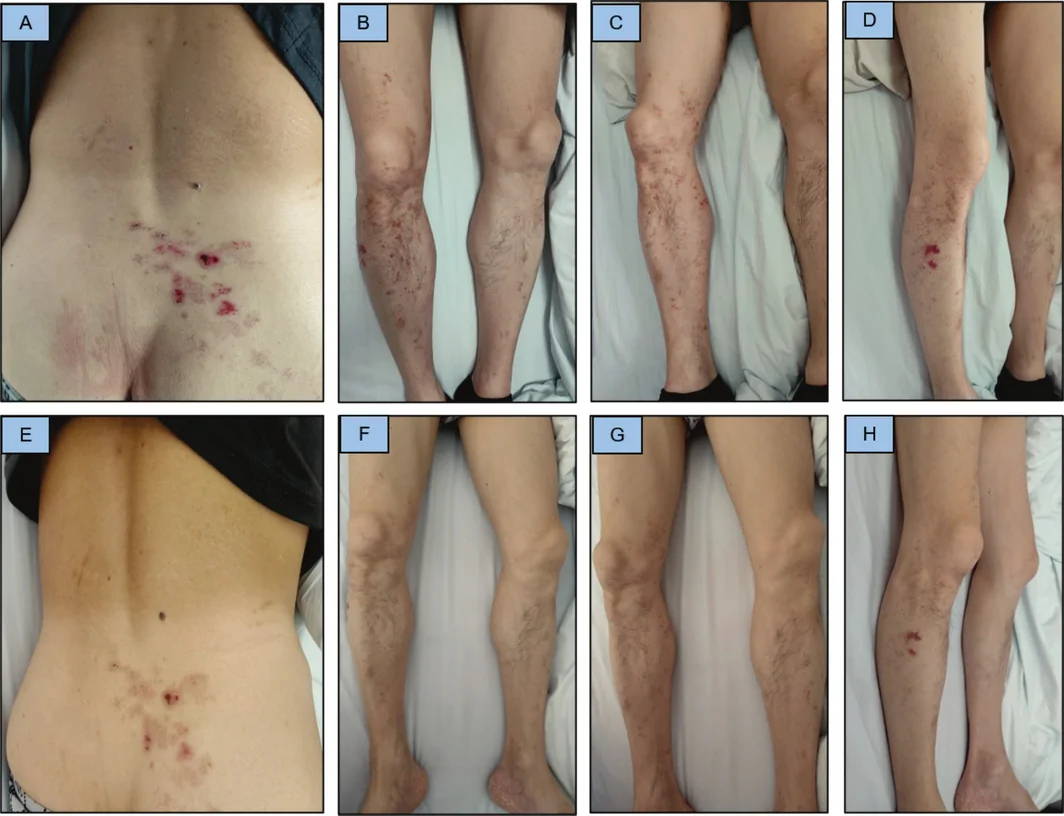
*Temporal evolution of the cutaneous Herpes Zoster eruption*. (A–D) Clinical photographs taken on Day 4 of admission, revealing grouped erythematous maculopapules and vesicles. The lesions are distributed along the right lumbar region, anterior thigh, and medial aspect of the lower leg. (E–H) Follow‐up images after 1 week of oral valaciclovir treatment, showing significant resolution of the rash with crust formation and decreased erythema.

0.3 g, orally (t.i.d.), was promptly started, alongside management for neuropathic pain (gabapentin, initiated at 0.1 g t.i.d. and titrated to effect). After 1 week of targeted therapy, the vesicles encrusted, and the lesions began to heal (Figure 2). At the 1‐month follow‐up, the rash had largely resolved; however, the patient exhibited mild Post‐Herpetic Neuralgia (PHN) within the L4 dermatome, characterized by mechanical allodynia (hypersensitivity to light touch).

At the 3‐month follow‐up, the patient demonstrated a favorable clinical outcome. The vesicular rash had completely crusted and healed with no signs of recurrence. Regarding postherpetic neuralgia (PHN), the patient reported that the neuropathic pain had essentially resolved, allowing for the discontinuation of analgesic medications. Furthermore, neurological recovery was satisfactory; the patient regained normal sensory function in the affected L4 dermatome without any residual motor weakness.

**FIGURE 3:**
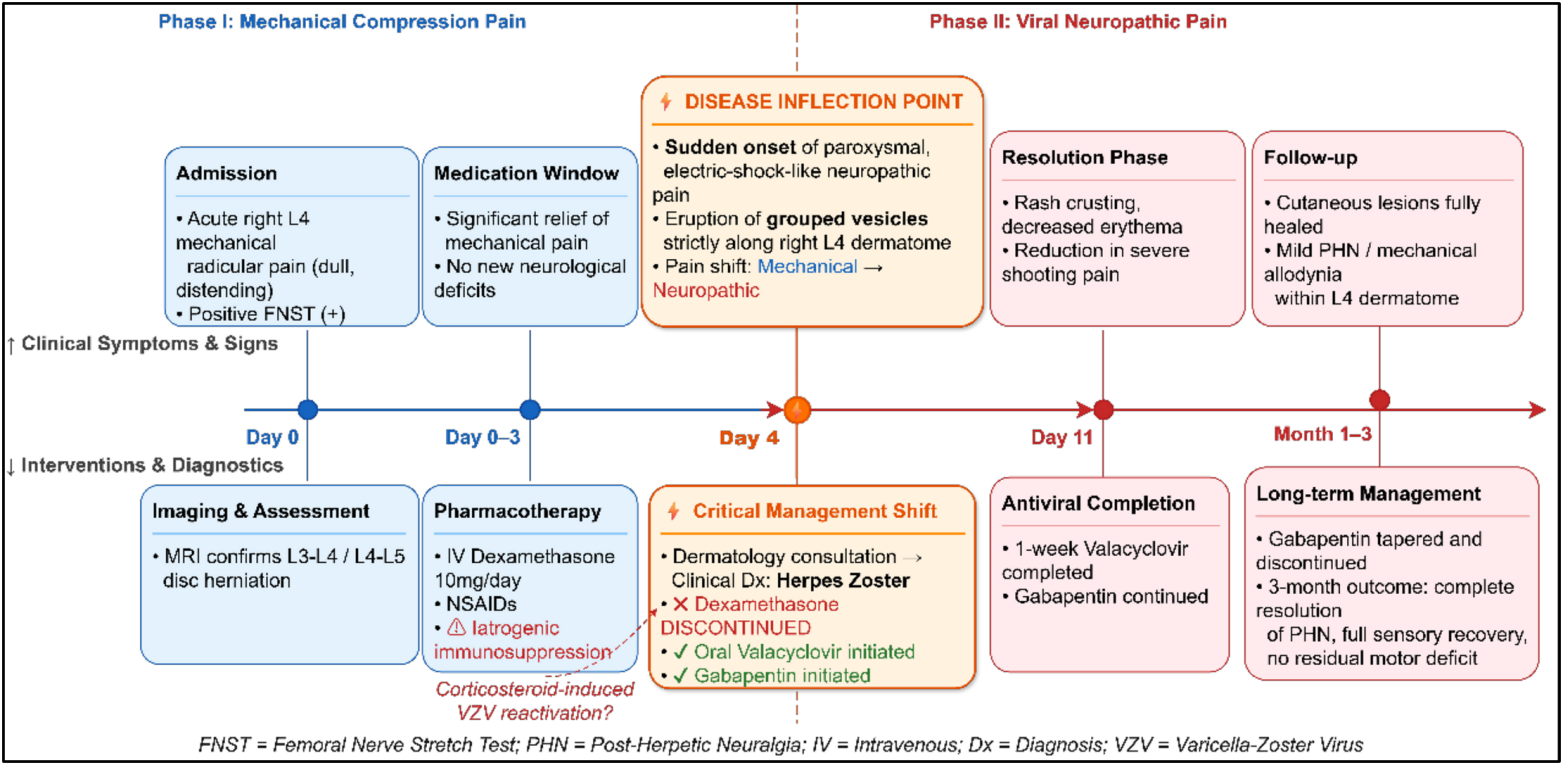
*Comprehensive timeline of clinical evolution and medical management*. The upper section illustrates the progression of clinical signs, highlighting the phenotypic transition from mechanical radicular pain to neuropathic pain concurrent with the vesicular eruption on Day 4. The lower section details the corresponding diagnostic and therapeutic sequence, notably the initial 3‐day course of intravenous dexamethasone, followed by an immediate management shift to antiviral and analgesic therapies upon VZV reactivation. FNST, Femoral Nerve Stretch Test; IV, Intravenous; NSAIDs, Non‐steroidal Anti‐inflammatory Drugs; Dx, Diagnosis; VZV, Varicella‐Zoster Virus; PHN, Post‐Herpetic Neuralgia.

A comprehensive timeline detailing the sequence of clinical symptoms, diagnostic evaluations, and therapeutic interventions, highlighting the phenotypic shift in pain following corticosteroid administration, is summarized in Figure 3.

## 3 Discussion

Lumbar disc herniation (LDH) represents a complex pathological entity underpinned by a profound local neuro‐immune inflammatory response [14, 15, 16, 17]. While systemic corticosteroids are sometimes utilized as a therapeutic option for radicular pain by dampening this inflammatory cascade [18, 19], their immunosuppressive nature risks triggering Varicella‐Zoster Virus (VZV) reactivation. This case illustrates how systemic corticosteroids might synergize with acute L4 nerve compression to alter the disease course, suggesting the hypothesis that structural neuropathy may create localized vulnerability for viral reactivation.

**FIGURE 4:**
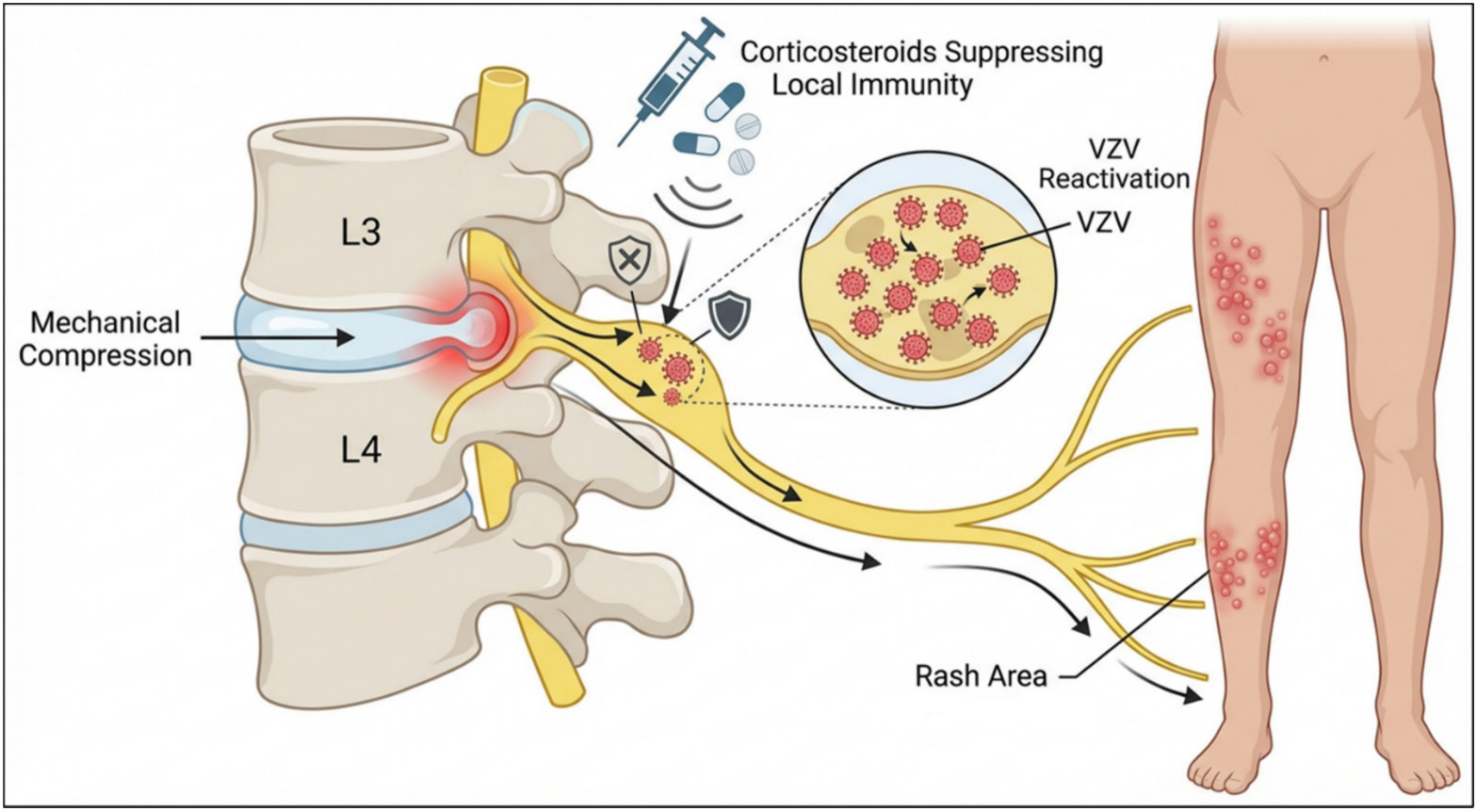
*Schematic representation of the “Immunocompromised District” (ICD) hypothesis illustrating the synergistic mechanism*. Chronic mechanical compression at the L3–L4 level may create a local vulnerability (microcirculatory impairment and neuro‐immune dysregulation). Systemic corticosteroid administration further suppresses cellular immunity, acting as a trigger. These local and systemic factors are hypothesized to facilitate VZV reactivation within the dorsal root ganglion. The reactivated virus then migrates via anterograde axonal transport along the sensory nerve fibers, resulting in the characteristic cutaneous eruption strictly confined to the compromised dermatome. This figure represents a hypothetical explanatory model based solely on the present clinical observations and should not be interpreted as demonstrating definitive biological causality.

The precise anatomical correlation between the structural compression and viral reactivation—with the vesicular rash strictly confined to the L4 dermatome—is highly consistent with the proposed “Immunocompromised District (ICD)” hypothesis [9]. We hypothesize that this phenomenon may be driven by a “double‐hit” process (Figure 4).

Systemic corticosteroids likely act as a precipitating “first hit” in this susceptible individual. By further attenuating cell‐mediated immunity, this pharmacologically induced dampening disrupts the already fragile immunological surveillance over latent VZV [20, 21]. However, the strict dermatomal confinement of the viral reactivation suggests a potential “second hit” associated with local mechanical vulnerability. Based on the biological plausibility derived from narrative literature, chronic physical impingement likely disrupts axoplasmic transport, impairs microcirculation, and alters local neuropeptide levels, thereby potentially rendering the compressed nerve root a locus minoris resistentiae. Ultimately, this clinical scenario underscores the core tenet of the “Immunocompromised District” theory, illustrating a potential scenario where the convergence of structural nerve compression and acute radicular inflammation engenders a localized immune vulnerability, which may contribute to a localized vulnerability that could help explain the anatomical distribution of the eruption.

To contextualize our findings and explicitly define the genuine novelty of this report, we have synthesized previously published cases concerning HZ associated with structural spinal pathology and corticosteroid administration (Table 1). Compared to the existing literature, the unique hypothesis‐generating value of our case lies in three main aspects.

**TABLE 1:** Published cases of herpes zoster associated with spinal pathology and corticosteroids versus the present case.

Category	References	Age/Sex	Initial presentation & imaging	HZ onset & interventions	Key findings/clinical lesson
**Scenario 1: HZ triggered by corticosteroids**	Hackenberg et al. [22]	62, M	Severe sciatica. MRI: slight spinal stenosis & swollen L5 root. Received local & epidural corticosteroid injections.	Vesicular rash erupted immediately after the epidural steroid infiltration.	Local or epidural immunosuppression from steroids for presumed structural pain can directly trigger VZV reactivation.
	Koda et al. [23]	74, M	Left sciatica for 1 month. MRI: L4/5 spinal canal stenosis. Received repeated caudal epidural steroid blocks.	Vesicular rash erupted on the foot after blocks failed, just as decompression surgery was considered.	Misattributing protracted pre‐eruptive HZ pain to MRI‐evident stenosis can lead to inappropriate epidural steroid injections.
**Scenario** **2: HZ neuropathy presenting as delayed/isolated motor weakness**	Choi et al. [24]	48, F	Leg pain mimicking sciatica followed by leg paresis.	Rash appeared **1 week after** the onset of leg paresis.	HZ (segmental zoster paresis) can perfectly mimic structural radiculopathy; EMG is crucial for localization.
	Patel et al. [25]	80, F	Severe L4 radiculopathy. MRI: no acute nerve root compression.	Leg weakness occurred **weeks after** the rash resolved.	Viral neuropathy can present solely as pain initially, with motor deficits appearing much later.
	Zhou et al. [26]	45, M	Left lower limb weakness presenting 2 months after full resolution of lumbar HZ rash.	Local hospital performed an unnecessary Head CT and discharged the patient without treatment.	The extremely prolonged latency between rash and myelitis‐induced paresis can severely misdirect diagnostic workup (e.g., scanning the brain instead of the spine).
**Scenario 3: HZ masked by concurrent disc herniation/stenosis**	Clavel [27]	44, F	Severe sciatica. Imaging: massive L4–L5 disc herniation; received conservative care.	Typical zoster rash erupted over the corresponding dermatome; underwent surgery.	Proposed that mechanical trauma/compression to the dorsal root ganglion from a herniated disc can precipitate VZV reactivation.
	Mallepally et al. [13]	30, M 45, F	Presented with sciatica. MRI confirmed real L3–4 and L4–5 disc bulges; misdiagnosed as discogenic radiculopathy.	Vesicular rash erupted **1 week** (Case 1) and **2 days** (Case 2) after initial presentation.	Asymptomatic or mild structural disc lesions on MRI can severely confound the diagnosis of pre‐eruptive HZ.
	Gao [28]	59, F	Left leg pain mimicking sciatica. CT showed multilevel disc protrusions; treated with lumbar traction.	Sporadic vesicles appeared **6 days** after symptom onset.	Incidental degenerative spine imaging findings often lead to misdirected mechanical treatments for early HZ.
	Fang et al. [29]	73, M	Left sciatica. MRI: L3–4 protrusion & L4–5 bulge. Initially misdiagnosed as discogenic sciatica.	Rash found on admission. Received antivirals and IM Diprospan. Severe motor paresis developed 1 week after rash resolved.	Concurrent disc bulges can mask pre‐eruptive HZ. Severe, delayed multisegmental paresis can still emerge despite antiviral and systemic steroid (Diprospan) therapy.
**Present Case**	Our case	52, M Comorbidity: Type 2 Diabetes	Acute severe radicular pain. MRI revealed a unique anatomical juxtaposition: an acute symptomatic L3–L4 herniation and a massive but quiescent chronic L4–L5 herniation.	Following systemic corticosteroid therapy, a vesicular rash erupted strictly confined to the L4 dermatome (correlating only with the acutely inflamed nerve root, completely sparing L5).	Three unique highlights: Anatomical: Acute neuroinflammation (L4) dictates local vulnerability, sparing chronic compression (L5). Etiological: “Double‐hit” trigger (systemic steroids + host diabetes/age). Clinical: Rapid pre‐eruptive pain shift (mechanical to neuropathic) as an early clue.

First, it provides a unique anatomical perspective. A deeper scrutiny of the radiologic Data reveals a compelling paradox: a dissociation between the static severity of structural degeneration and the pattern of viral reactivation. Although MRI demonstrated severe chronic L4–L5 disc degeneration without focal root compression, the viral outbreak concentrated within the L4 dermatome, corresponding perfectly to the acute symptomatic L3–L4 root compression. The imposing L4–L5 herniation remained clinically quiescent, indicating a chronic lesion with physiological accommodation. Based purely on the rapid onset and severity of clinical symptoms, we speculate that the acute L3–L4 presentation likely involved not only static mechanical impingement but also significant acute chemical radiculitis, driven by the local secretion of inflammatory cytokines (e.g., TNF‐α, IL‐1β) [8, 30]. We hypothesize that acute neuro‐inflammation may disrupt local neuro‐immune homeostasis more profoundly than chronic mechanical impingement, potentially creating a favorable microenvironment for VZV reactivation. This clinical disparity suggests that acute neuroinflammation, rather than static mechanical pressure alone, might contribute to segmental immune vulnerability, raising the possibility that acute radiculopathy could serve as a localized predisposing factor for zoster.

Second, it highlights a potential multifactorial trigger. Importantly, the “double‐hit” mechanism proposed in this report remains entirely speculative. A major limitation of our case report is the absence of direct, objective biological evidence. We did not conduct measurements of local immune activity, inflammatory cytokine levels, VZV viral load, dorsal root ganglion pathology, or immune cell function. Therefore, our pathophysiological explanations should be interpreted strictly as a hypothesis requiring future experimental validation. Importantly, we must exercise caution in attributing strict causality solely to corticosteroid administration. The pathogenesis of HZ in this patient is undoubtedly multifactorial. The patient possessed significant baseline risk factors for viral reactivation, primarily age‐related immunosenescence (age > 50 years) and a history of Type 2 Diabetes Mellitus, both of which independently impair cell‐mediated immunity [31, 32]. Given these preexisting vulnerabilities and the ubiquitous latency of VZV following childhood varicella, the possibility of spontaneous reactivation or coincidental occurrence cannot be definitively excluded. Rather than acting as the sole trigger, we propose that the systemic corticosteroids, superimposed on the localized acute neuroinflammation, likely functioned as the final precipitating factors—a “tipping point”—that breached the already compromised immunological threshold in this susceptible individual.

**FIGURE 5:**
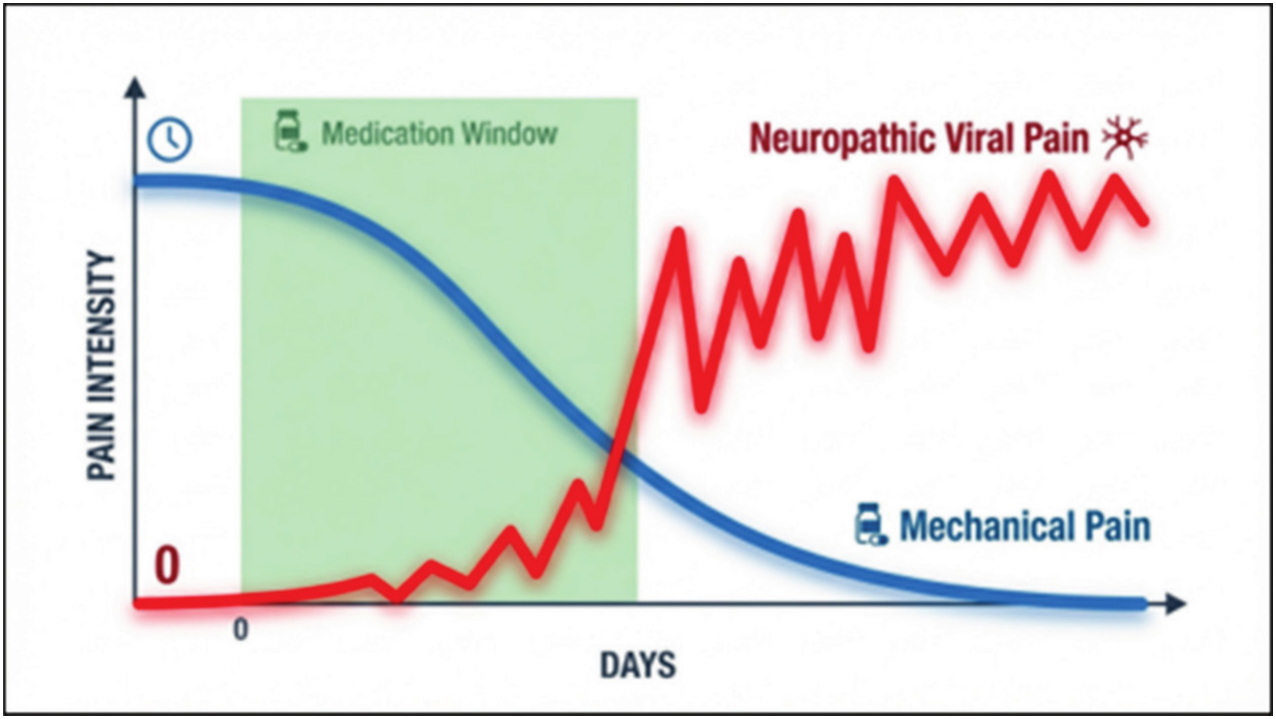
*Schematic illustration of the temporal “Pain Phenotype Transformation*.” The blue trajectory represents the initial mechanical compressive pain (characterized by a continuous dull ache), which gradually subsided following the administration of corticosteroids during the medication window (green shaded area). Conversely, the red jagged curve depicts the subsequent emergence and escalation of neuropathic viral pain (characterized by paroxysmal, electric‐shock‐like sensations). The intersection of these two curves illustrates the “Crossover Phenomenon”: As the anti‐inflammatory effect masks the mechanical symptoms, the immunosuppressive effect triggers the viral rebound, leading to a complete shift in pain etiology.

*Third, we document a specific semi‐logical evolution as an early clinical clue*. Clinically, the overlapping presentation created a diagnostic challenge. The radicular pain, supported by a positive Femoral Nerve Stretch Test (FNST), likely confounded the early clinical picture and masked the subtle prodromal signs of viral ganglionitis. In such complex cases, relying solely on standard orthopedic maneuvers may not always be sufficient to distinguish the underlying etiology of neuropathic pain. This case highlights that closely monitoring for a shift in pain characteristics (a phenotypic change in pain presentation, Figure 5) can serve as a valuable clinical clue. We observed a distinct qualitative shift in pain presentation, transitioning from a mechanical “dull, distending ache” to a “burning or electric‐shock‐like” neuropathic pain characteristic of viral reactivation. Failing to recognize this sensory transition and inappropriately escalating corticosteroid therapy [33] may further compromise local immunity, potentially precipitating a severe viral eruption.

Although targeted antivirals resolved the cutaneous manifestations, the patient developed significant mechanical allodynia—a hallmark of central sensitization. We suggest a potential association: high‐dose steroids during the acute phase might have facilitated rapid viral replication. This intense viral activity potentially inflicts profound structural damage upon the sensory nerve architecture, suggesting that the short‐term relief provided by corticosteroids may compromise long‐term neural functional integrity.

Consequently, we suggest increased clinical vigilance and individualized risk stratification when managing lumbar disc herniation with systemic corticosteroids. For potentially vulnerable patients, reviewing varicella exposure history could be a valuable step prior to therapy. Clinicians should closely monitor symptom evolution; an unexpected qualitative shift toward neuropathic pain might serve as an early prodromal warning of HZ. Upon identifying such sensory transitions, a high index of clinical suspicion and frequent dermatological examinations are crucial to initiate antivirals promptly once the rash manifests. Regarding corticosteroid management, the rapid onset of HZ in this patient highlights the necessity for careful, individualized risk–benefit assessments. While prompt tapering or cessation of corticosteroids was deemed necessary in this specific case to mitigate further immunosuppression, such decisions should be made cautiously on a case‐by‐case basis. Additionally, gabapentin was utilized in this instance strictly for the symptomatic management of acute herpetic neuropathic pain. Crucially, a major limitation of this report is the complete reliance on clinical and macroscopic observations without objective laboratory or radiological validation. As astutely pointed out, we lack measurements of local immune activity, cytokines, and VZV viral load. Consequently, the diagnosis of VZV reactivation relies entirely on a clinical dermatological evaluation without gold‐standard virological verification (e.g., PCR). Furthermore, we did not perform contrast‐enhanced MRI, nerve ultrasound, or electromyography to definitively quantify the local structural inflammation. Therefore, our assertion that acute radicular inflammation creates a localized immune vulnerability remains strictly a hypothesis. Future studies incorporating these objective diagnostic modalities are essential to validate the proposed neuro‐immunological mechanisms. We acknowledge that the primary limitation of this report is its single‐case design; therefore, the proposed ‘double‐hit’ mechanism requires further validation in larger clinical cohorts, and the specific management steps taken in this instance should not be extrapolated into generalized clinical recommendations.

## 4 Conclusions

This case supports a hypothesis of a potential “double‐hit” mechanism in which systemic immunosuppression associated with corticosteroid therapy may interact with localized neuro‐immune vulnerability related to acute radiculopathy. Clinicians must remain vigilant regarding the phenotypic shift of pain—from mechanical to neuropathic—in patients undergoing steroid therapy. Recognizing this dynamic is paramount to avoid misinterpreting viral prodromes as mechanical exacerbations, thereby ensuring timely antiviral intervention.

## 5 Patient Perspective

“Initially, I thought my back and leg pain was simply getting worse despite the steroid treatment, and the sudden, electric‐shock‐like pain was terrifying. I was deeply concerned when the rash appeared. However, once the doctors explained the true cause—a viral infection triggered by the medication and my nerve compression—and started the antiviral treatment, the severe pain subsided significantly. I am grateful for their accurate diagnosis and am satisfied with my recovery progress.”

## Author Contributions

**Jun Liu:** conceptualization, methodology. **Lili Luo:** software, data curation. **Ming Zhou:** investigation, validation. **Weifeng Chen:** formal analysis, supervision. **Na Cao:** supervision, formal analysis. **Zhanglin Pu:** visualization, project administration. **Yunwei Niu:** validation, formal analysis. **Jiachen Guo:** visualization, project administration. **Huayuan Liao:** project administration, visualization, writing – original draft. **Yi Li:** writing – original draft, formal analysis. **Jun Gao:** writing – review and editing, funding acquisition. **Kang Wei:** writing – review and editing.

## Funding

This project is funded by the Natural Science Foundation of Jiangsu Province (General Program), BK20211066.

## Ethics Statement

Written informed consent to participate was obtained from the patient. The ethical approval for reporting this case was granted by the Ethics Committee of Changzhou Hospital Affiliated to Nanjing University of Chinese Medicine. All procedures performed in studies involving human participants were in accordance with the ethical standards of the institutional and/or national research committee and with the 1964 Helsinki declaration and its later amendments or comparable ethical standards.

## Consent

Written informed consent was obtained from the patient for publication of this case report and any accompanying images. A copy of the written consent is available for review by the Editor‐in‐Chief of this journal.

## Conflicts of Interest

The authors declare no conflicts of interest.

## Data Availability

All data generated or analyzed during this study are included in this published article.
